# Complications Following Elective Major Noncardiac Surgery Among Patients With Prior SARS-CoV-2 Infection

**DOI:** 10.1001/jamanetworkopen.2022.47341

**Published:** 2022-12-16

**Authors:** Kieran L. Quinn, Anjie Huang, Chaim M. Bell, Allan S. Detsky, Lauren Lapointe-Shaw, Laura C. Rosella, David R. Urbach, Fahad Razak, Amol A. Verma

**Affiliations:** 1 Department of Medicine, University of Toronto, Toronto, Ontario, Canada; 2 ICES, Toronto, Ontario, Canada; 3 ICES, Ottawa, Ontario, Canada; 4 Institute of Health Policy, Management and Evaluation, University of Toronto, Toronto, Ontario, Canada; 5 Department of Medicine, Sinai Health System and University Health Network, Toronto, Ontario, Canada; 6 Temmy Latner Centre for Palliative Care, Toronto, Ontario, Canada; 7 Women’s College Hospital, Women’s College Research Institute, Departments of Surgery and Health Policy, Management and Evaluation, University of Toronto, Toronto, Ontario, Canada; 8 Li Ka Shing Knowledge Institute, Department of Medicine, Unity Health Toronto, Toronto, Ontario, Canada; 9 Department of Medicine, St Michael’s Hospital, Unity Health Toronto, Toronto, Ontario, Canada

## Abstract

**Importance:**

There is an urgent need for evidence to inform preoperative risk assessment for the millions of people who have had SARS-CoV-2 infection and are awaiting elective surgery, which is critical to surgical care planning and informed consent.

**Objective:**

To assess the association of prior SARS-CoV-2 infection with death, major adverse cardiovascular events, and rehospitalization after elective major noncardiac surgery.

**Design, Setting, and Participants:**

This population-based cohort study included adults who had received a polymerase chain reaction test for SARS-CoV-2 infection within 6 months prior to elective major noncardiac surgery in Ontario, Canada, between April 2020 and October 2021, with 30 days follow-up.

**Exposures:**

Positive SARS-CoV-2 polymerase chain reaction test result.

**Main Outcomes and Measures:**

The main outcome was the composite of death, major adverse cardiovascular events, and all-cause rehospitalization within 30 days after surgery.

**Results:**

Of 71 144 patients who underwent elective major noncardiac surgery (median age, 66 years [IQR, 57-73 years]; 59.8% female), 960 had prior SARS-CoV-2 infection (1.3%) and 70 184 had negative test results (98.7%). Prior infection was not associated with the composite risk of death, major adverse cardiovascular events, and rehospitalization within 30 days of elective major noncardiac surgery (5.3% absolute event rate [n = 3770]; 960 patients with a positive test result; adjusted relative risk [aRR], 0.91; 95% CI, 0.68-1.21). There was also no association between prior infection with SARS-CoV-2 and postoperative outcomes when the time between infection and surgery was less than 4 weeks (aRR, 1.15; 95% CI, 0.64-2.09) or less than 7 weeks (aRR, 0.95; 95% CI, 0.56-1.61) and among those who were previously vaccinated (aRR, 0.81; 95% CI, 0.52-1.26).

**Conclusions and Relevance:**

In this study, prior infection with SARS-CoV-2 was not associated with death, major adverse cardiovascular events, or rehospitalization following elective major noncardiac surgery, although low event rates and wide 95% CIs do not preclude a potentially meaningful increase in overall risk.

## Key Points

**Question:**

Is prior infection with SARS-CoV-2 associated with death, major adverse cardiovascular events, and rehospitalization after elective major noncardiac surgery?

**Findings:**

This population-based cohort study of 960 people with prior SARS-CoV-2 infection and 70 184 controls with negative test results did not find an association between prior COVID-19 and death, major adverse cardiovascular events, or rehospitalization within 30 days after elective major noncardiac surgery.

**Meaning:**

Prior infection with SARS-CoV-2 was not associated with adverse outcomes following elective major noncardiac surgery, although low event rates and wide 95% CIs do not preclude a potentially meaningful increase in overall risk.

## Teaser

This cohort study assesses the association of prior SARS-CoV-2 infection with death, major adverse cardiovascular events, and rehospitalization after elective major noncardiac surgery.

## Introduction

To date, there have been over 630 million documented cases of SARS-CoV-2 infection worldwide, resulting in elective surgery delays for tens of millions of people.^1,2^ This massive global backlog means that millions of people awaiting elective surgery are also likely to have had SARS-CoV-2 infection. SARS-CoV-2 infection is associated with increased risk of cardiovascular disease and venous thromboembolism, and many people who survive infection with SARS-CoV-2 have reduced functional reserve and are at increased risk of death.^3,4,5,6^ SARS-CoV-2 infection may therefore be associated with increased risk of adverse outcomes following elective surgery.^7^

There is an urgent need for evidence to inform preoperative risk assessment, as it is critical to surgical care planning and to informing patient consent. Several prior studies^8,9,10,11,12,13^ found that SARS-CoV-2 infection prior to elective or emergency surgery was associated with a greater risk of adverse outcomes. However, these studies were conducted in the prevaccination era, examined pulmonary complications, and included only a small number of patients undergoing elective surgery. These studies lacked population-level data, measurement of cardiovascular outcomes, data on the effects of vaccination, and data on the effects of timing of elective surgery relative to infection with SARS-CoV-2. The objective of this population-level cohort study was to assess the association of prior SARS-CoV-2 infection with death, major adverse cardiovascular events (MACE), and all-cause rehospitalization within 30 days of elective major noncardiac surgery among all adults with prior testing for SARS-CoV-2 within 6 months before surgery.

## Methods

### Study Design, Setting, and Data Sources

This study is reported in accordance with guidelines for the Reporting of Studies Conducted Using Observational Routinely-Collected Health Data (RECORD).^14^ We conducted a population-based cohort study in Ontario, Canada, using linked clinical and health administrative databases. These validated databases are widely used to conduct population-level studies in Ontario (eAppendix in Supplement 1). Using a comparator group of people who tested negative for SARS-CoV-2 prior to surgery has the advantage of minimizing confounding and selection bias related to health care–seeking behaviors and misclassification of exposure status.^15^ The administrative data sets used in this study were linked using encoded identifiers at the patient level and analyzed at ICES (formerly the Institute of Clinical and Evaluative Sciences). Ontario is Canada’s most populous province, with over 13 million adults and more than 1 million cases of SARS-CoV-2 infection. Residents of Ontario have public access to hospital care and physicians’ services, and those aged 65 years or older are provided prescription drug insurance coverage. The use of data in this project was authorized under section 45 of Ontario’s Personal Health Information Protection Act, which does not require review by a research ethics board and authorizes ICES to collect personal health information, without consent, for the purpose of analysis or compiling statistical information with respect to the management, evaluation, or monitoring of the allocation of resources to or planning for all or part of the health system.

### Study Cohort

Our study cohort included all Ontario adults (aged ≥18 years) who underwent elective major noncardiac surgery between April 2020 and October 2021 and had polymerase chain reaction (PCR) SARS-CoV-2 testing within 6 months prior to surgery. The 6-month exposure period was intentionally chosen given emerging evidence about long-term effects of SARS-CoV-2 infection.^16,17^ We performed 2 additional analyses, stratifying the sample by patients with SARS-CoV-2 infection less than 4 weeks and less than 7 weeks prior to surgery, based on a prior study finding that adverse outcomes were most frequently observed within these windows.^13^

The index date was the day of surgery. We excluded people who underwent emergency surgery, had missing data on age or sex at index date, were younger than 18 years or older than 105 years, were nonresidents of Ontario, were not eligible for the Ontario Health Insurance Plan for a period of 3 months or more in the year prior to the index date, and did not have at least 1 physician visit in 10 years or more prior to the index date. We selected surgeries that had greater than 1% risk of MACE and were among the most common surgeries performed in Ontario (Table 1).^18,19,20^

**Table 1.** Baseline Characteristics of Patients Who Underwent Elective Major Noncardiac Surgery in Ontario Between April 2020 and October 2021 According to the Results of Their SARS-CoV-2 Test Completed Within 6 Months of Surgery

Characteristic	Patients^a^		Standardized difference
	With prior SARS-CoV-2 infection (n = 960)	Without prior SARS-CoV-2 infection (n = 70 184)	
Age, median (IQR)	61 (52-69)	66 (57-73)	0.33
Sex			
Female	598 (62.3)	42 211 (60.1)	0.04
Male	362 (37.7)	27 973 (39.9)	0.04
Rural residence	70 (7.3)	10 317 (14.7)	0.24
Neighborhood income quintile			
1	195-199 (20.3-20.7)^b^	12 305 (17.5)	0.08
2	204 (21.3)	13 611 (19.4)	0.05
3	223 (23.2)	14 252 (20.3)	0.07
4	181 (18.9)	14 399 (20.5)	0.04
5	152 (15.8)	15 462 (22.0)	0.16
Missing	1-10 (0.0-0.2)^b^	155 (0.2)	0
Ethnicity^c^			
Chinese	16-20 (1.7-2.1)^b^	1629 (2.3)	0.02
South Asian	72 (7.5)	1797 (2.6)	0.23
General population	867 (90.3)	66 741 (95.1)	0.18
Missing	1-10 (0.0-0.2)^b^	17 (0.0)	0.03
Vaccination status			
Full	266 (27.7)	14 369 (20.5)	0.17
Partial	180 (18.8)	7520 (10.7)	0.23
Unvaccinated	514 (53.5)	48 295 (68.8)	0.32
Time from COVID test to surgery, d			
Median (IQR)	88 (46-136)	3 (3-5)	1.59
Mean (SD)	89.21 (52.99)	11.92 (29.58)	1.80
RCRI			
0	59 (6.1)	3478 (5.0)	0.05
1	635 (66.1)	46 930 (66.9)	0.02
2	216 (22.5)	16 011 (22.8)	0.01
3	43 (4.5)	3131 (4.5)	0
≥4	7 (0.7)	634 (0.9)	0.02
Chronic conditions			
Asthma	133 (13.9)	12 025 (17.1)	0.09
Arrhythmia	40 (4.2)	4110 (5.9)	0.08
Cancer	337 (35.1)	25 797 (36.8)	0.03
Chronic kidney disease	58 (6.0)	3468 (4.9)	0.05
Cirrhosis (decompensated)	18 (1.9)	867 (1.2)	0.05
Coronary artery disease	64 (6.7)	6027 (8.6)	0.07
COPD prevalent	42 (4.4)	4054 (5.8)	0.06
Dementia	8 (0.8)	781 (1.1)	0.03
Diabetes	254 (26.5)	16 282 (23.2)	0.08
Heart failure	37 (3.9)	3107 (4.4)	0.03
Hypertension	484 (50.4)	38 335 (54.6)	0.08
Osteoarthritis	508 (52.9)	41 574 (59.2)	0.13
Stroke	23 (2.4)	1249 (1.8)	0.04
Venous thromboembolism	17 (1.8)	1118 (1.6)	0.01
Hospital Frailty Score			
0	180 (18.8)	14 124 (20.1)	0.03
0.1-4.9	142 (14.8)	9520 (13.6)	0.04
5.0-8.9	35 (3.6)	2206 (3.1)	0.03
≥9.0	39 (4.1)	1756 (2.5)	0.09
Not hospitalized	564 (58.8)	42 578 (60.7)	0.04
Resided in nursing home	1-10 (0.0-0.2)^b^	132 (0.2)	0
Non–COVID-related rehospitalization in 1 y prior to surgery	164 (17.1)	12 101 (17.2)	0
Hospital annual surgery volume, tertile			
0	9 (0.9)	1085 (1.5)	0.05
1	167 (17.4)	13 727 (19.6)	0.06
2	766 (79.8)	54 310 (77.4)	0.06
Missing	18 (1.9)	1062 (1.5)	0.03
Surgery at teaching hospital	271 (28.2)	22 369 (31.9)	0.08
Type of surgery			
Abdominal aortic aneurysm repair	9 (0.9)	1053 (1.5)	0.05
Carotid endarterectomy	12 (1.3)	778 (1.1)	0.01
Total and radical cystectomy	9 (0.9)	624 (0.9)	0.01
Gastrectomy or esophagostomy	96 (10.0)	4117 (5.9)	0.15
Total hip replacement	82 (8.5)	11 163 (15.9)	0.23
Hysterectomy	221 (23.0)	13 204 (18.8)	0.1
Total knee replacement	291 (30.3)	22 449 (32.0)	0.04
Partial liver resection	11 (1.1)	850 (1.2)	0.01
Partial or total nephrectomy	47 (4.9)	2269 (3.2)	0.08
Pneumonectomy and lobectomy	25 (2.6)	1676 (2.4)	0.01
Radical prostatectomy	25 (2.6)	2001 (2.9)	0.02
Peripheral vascular surgery	15 (1.6)	1532 (2.2)	0.05
Large bowel and rectal surgery	107 (11.1)	7818 (11.1)	0
Whipple	10 (1.0)	650 (0.9)	0.01

Abbreviations: COPD, chronic obstructive pulmonary disease; RCRI, Revised Cardiac Risk Index.

^a^ Data are presented as number (percentage) of patients unless otherwise indicated.

^b^ Suppressed due to small cells.

^c^ This data set was derived by applying validated lists of South Asian and Chinese surnames to the raw Registered Persons Database, which includes surnames to assign an ethnicity to all Ontario residents. The “general population” includes all individuals whose surname was not on either list, including those from other visible minority groups. Each individual’s earliest known surname is used to assign ethnicity to avoid misclassification due to name changes at marriage.

### Patient and Hospital Characteristics

We measured multiple validated demographic and clinical variables, including age; sex; socioeconomic status; residence in a nursing home; rural location of residence; ethnicity (Chinese, South Asian, and general population);^21^ comorbidities;^22^ Hospital Frailty Risk Score;^23^ prior hospitalization and intensive care unit admission for COVID-19; preoperative COVID-19 vaccination status; preoperative consultation with anesthesia, internal medicine, and cardiology; use of preoperative testing, including echocardiography, noninvasive cardiac stress testing, and percutaneous coronary angiography; and Revised Cardiac Risk Index Score (RCRI). The RCRI is an externally validated tool that predicts major operative cardiovascular complications at 30 days for patients undergoing elective noncardiac procedures.^19,24,25^ Ethnicity was included to describe potential differences in the characteristics of people who did and did not have a test positive for SARS-CoV-2 that may also be associated with differential risk of outcomes. This data set was derived by applying validated lists of South Asian and Chinese surnames to the raw Registered Persons Database, which includes surnames to assign an ethnicity to all Ontario residents. The “general population” includes all individuals whose surname was not on either list, including those from other visible minority groups. Each individual’s earliest known surname is used to assign ethnicity to avoid misclassification due to name changes at marriage. Full vaccination status was defined as having received 2 doses of approved vaccine, and partial status was defined as having received 1 dose, which corresponded to the established provincial criteria during the study period, before the availability of third and fourth booster doses. We determined a hospital’s annual surgical volume as well as its status as an academic or community hospital.

### Prior SARS-CoV-2 Infection

We determined the presence of prior SARS-CoV-2 infection based on a confirmed positive PCR test result within 6 months of surgery. In Ontario, most patients undergoing surgery had routine preoperative testing for SARS-CoV-2 infection; results from all PCR tests are reported in a provincial database. If a person tested positive for SARS-CoV-2 within 6 months of their surgery, we also determined the time from their positive test result to their date of surgery. For people without a prior positive test result during this interval (including those who may have tested positive longer than 6 months prior to surgery), we reported time from their most recent negative test result to surgery.

### Outcomes

The primary outcome was a composite of death, MACE, and all-cause rehospitalization within 30 days. MACE was defined as acute myocardial infarction, unstable angina, transient ischemic attack, stroke, heart failure, and cardiovascular death. The secondary outcomes were the individual components of the primary outcome.

### Statistical Analysis

Multivariable modified Poisson regression measured the association of prior SARS-CoV-2 infection with the composite of death, MACE, and all-cause rehospitalization within 30 days. An analysis accounting for the competing risk of death was not performed because the absolute percentage of people who died was low and therefore unlikely to bias the overall results.^26,27^ The use of a modified Poisson regression allows estimation of relative risk (RR) over odds ratios, which are often less intuitive to the practicing clinician.^28^

To examine factors that may modify the association of SARS-CoV-2 infection with surgical risk, we stratified the primary analysis by (1) timing of surgery relative to the test positive for SARS-CoV-2 (<4 vs ≥4 weeks, <7 vs ≥7 weeks) because prior research demonstrated each 4-week delay in surgery for patients with cancer was associated with increased risk of death by approximately 10%^13,29,30^ and operative risk may return to baseline risk at 7 weeks and beyond; (2) vaccination status; (3) RCRI score, and (4) surgery type, which we categorized as a combination of hip and knee replacements vs all others. We also examined the associated risk of the primary outcome among those with prior SARS-CoV-2 infection who had surgery less than 7 weeks vs 7 weeks or more and less than 4 weeks vs 4 weeks of more from a person’s positive test result. We explored the possibility of selection bias by examining the baseline characteristics of patients who underwent elective major noncardiac surgery and were tested for SARS-CoV-2 prior to surgery compared with those of patients who were not tested. All analyses were performed using SAS, version 9.4 (SAS Institute Inc).

## Results

### Baseline Characteristics

**Figure 1.**
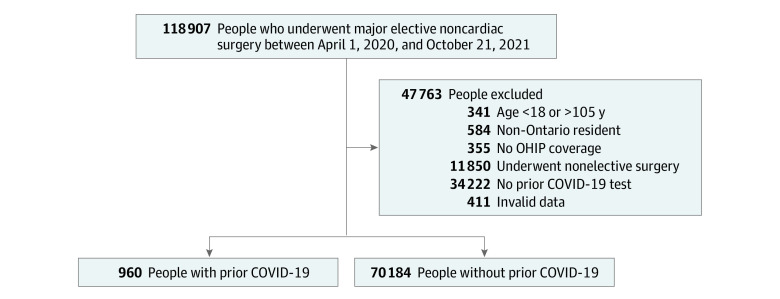
Flowchart of the Study Cohort

There were 71 144 patients who underwent elective major noncardiac surgery during the study period, 960 (1.3%) of whom had a positive PCR result for SARS-CoV-2 and 70 184 (98.7%) of whom has a negative test result in the 6 months prior to surgery (Figure 1). The median age was 66 years (IQR, 57-73 years); 59.8% were female, and 40.2% were male (Table 1). The majority of the cohort (66.4%) had an RCRI score of 1, corresponding to a predicted 30-day risk of major postoperative cardiovascular complications of 6.0% (95% CI, 4.9%-7.4%).^24,25^ The overall rate of full or partial vaccination prior to surgery among the cohort was 31.1%. The median time from test to surgery was 88 days (IQR, 46-136 days) among people with prior SARS-CoV-2 infection and 3 days (IQR 3-5 days) among those who tested negative. The baseline characteristics of patients who underwent elective major noncardiac surgery and were tested for SARS-CoV-2 prior to surgery compared with those who were not tested were generally similar (Table 1).

### Postoperative Outcomes

**Figure 2.**
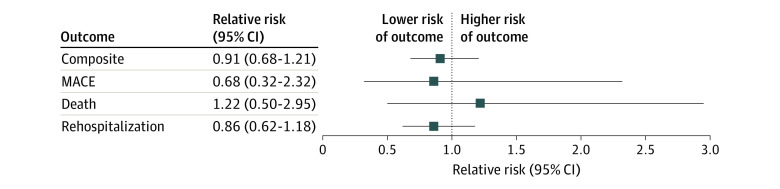
Risk of Death, Major Adverse Cardiac Events (MACE), and Rehospitalization Within 30 Days After Elective Major Noncardiac Surgery Among Patients Who Tested Positive for SARS-CoV-2 Within 6 Months Before Surgery Data are from patients who underwent surgery in Ontario between April 2020 and October 2021.

**Figure 3.**
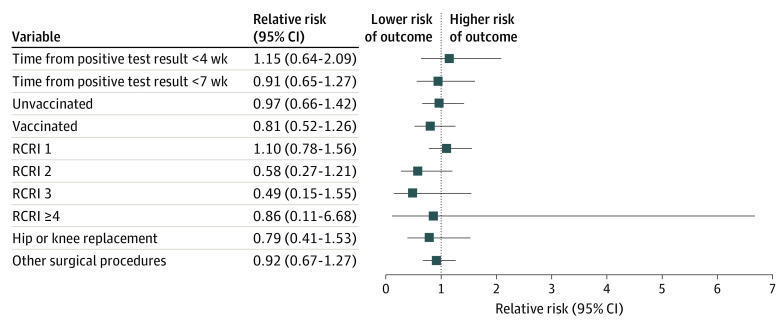
Risk of Death, Major Adverse Cardiac Events (MACE), and Rehospitalization Within 30 Days After Elective Major Noncardiac Surgery Among Patients Who Tested Positive for SARS-CoV-2 Within 6 Months Before Surgery, Stratified by Potential Modifying Factors Data are from patients who underwent surgery in Ontario between April 2020 and October 2021, stratified by time from test to surgery, vaccination status, Revised Cardiac Risk Index Score (RCRI) score, and surgery type. An RCRI of 0 is not reported, as the model would not converge.

The overall composite outcome of death, MACE, and all-cause rehospitalization within 30 days of surgery occurred in 5.3% of the cohort (n = 3770). After adjustment, prior SARS-CoV-2 infection was not associated with the composite risk of death, MACE, and rehospitalization within 30 days of elective major noncardiac surgery (960 patients with a positive test result; adjusted RR [aRR], 0.91; 95% CI, 0.68-1.21) or with any of the individual components of the composite primary outcome (Figure 2 and Table 2). Prior SARS-CoV-2 infection was not associated with the composite outcome when stratifying the analyses by preoperative risk according to the RCRI score (RCRI 1: 635 patients with a positive test result; aRR, 1.10 [95% CI, 0.78-1.56]; RCRI 2: 216 patients with a positive test result; aRR, 0.58 [95% CI, 0.27-1.21]; RCRI 3: 43 patients with a positive test result; aRR, 0.49 [95% CI, 0.15-1.55]; RCRI≥4: 7 patients with a positive test result; aRR, 0.86 [95% CI, 0.11-6.68]) or by surgery type (hip and knee replacement: 373 patients with a positive test result; aRR, 0.79 [95% CI, 0.41-1.53]; all other surgeries: 587 patients with a positive test result; aRR, 0.92 [95% CI, 0.67-1.27]) (Figure 3).

**Table 2.** Crude Event Rate and Unadjusted and Adjusted Risk Of Death, MACE, and Rehospitalization Within 30 Days After Elective Major Noncardiac Surgery Among Patients With Prior SARS-Cov-2 Infection Compared With Controls Without Prior SARS-Cov-2 Infection^a^

Outcome	Crude event rate, No. (%)		Relative risk (95% CI)	
	Positive PCR result (n = 960)	Negative PCR result (n = 70 184)	Unadjusted	Adjusted^b^
Composite of death, MACE, and rehospitalization	47 (4.9)	3723 (5.3)	0.92 (0.69-1.23)	0.91 (0.68-1.21)
Death	5 (0.5)	344 (0.5)	1.06 (0.44-2.57)	1.22 (0.50-2.95)
MACE	4 (0.4)	371 (0.5)	0.79 (0.29-2.11)	0.86 (0.32-2.23)
Rehospitalization	39 (4.1)	3212 (4.6)	0.89 (0.65-1.22)	0.86 (0.62-1.18)

Abbreviations: COPD, chronic obstructive pulmonary disease; MACE, major adverse cardiovascular events; PCR, polymerase chain reaction.

^a^ Data are from patients who underwent elective major noncardiac surgery in Ontario between April 2020 and October 2021.

^b^ Model was adjusted for age, sex, rural residence, neighborhood income quintile, type of surgery, history of asthma, arrhythmia, cancer, chronic kidney disease, decompensated cirrhosis, coronary artery disease, COPD, dementia, diabetes, heart failure, hypertension, stroke, nursing home residence at the time of surgery, Hospital Frailty Risk Score categories, vaccination status, number of hospitalizations in the year prior to surgery not related to COVID-19, and categories of the Revised Cardiac Risk Index score.

### Stratification by Timing of Surgery and Preoperative Vaccination Status

Prior infection was not associated with adverse postoperative outcomes within 30 days of surgery when stratifying by the timing of surgery of less than 7 weeks (254 patients with a positive test result, aRR, 0.95; 95% CI, 0.56-1.61) and 7 weeks or more (706 patients with a positive test result; aRR, 0.89; 95% CI, 0.63-1.26) from their test positive for SARS-CoV-2 compared to those without prior SARS-CoV-2 infection or when directly comparing the timing of surgery of less than 7 vs 7 weeks or more among those with prior SARS-CoV-2 infection (aRR, 1.28; 95% CI, 0.61-2.67) (Figure 3). Prior infection with SARS-CoV-2 was not associated with the composite primary outcome when stratifying by the timing of surgery of less than 4 weeks (149 patients with a positive test result; aRR, 1.15; 95% CI, 0.64-2.09) and 4 weeks or more (811 patients with a positive test result; aRR, 0.85; 95% CI, 0.61-1.19) from their test positive for SARS-CoV-2 compared with those without prior SARS-CoV-2 infection or when directly comparing the timing of surgery of less than 4 vs 4 weeks or more among those with prior SARS-CoV-2 infection (149 without prior SARS-CoV-2 infection vs 811 patients with a positive test result; aRR, 1.70; 95% CI, 0.77-3.76) (Figure 3).

Among people who were partially and fully vaccinated against COVID-19, there was no association between prior SARS-CoV-2 infection and postoperative outcomes (446 patients with a positive test result; aRR, 0.81; 95% CI, 0.52-1.26) (Figure 3). Similarly, there was no association of preoperative vaccination status with the primary outcome among fully vaccinated people (266 patients with a positive test result; aRR, 0.89; 95% CI, 0.51-1.55) and among unvaccinated people (514 patients with a positive test result; aRR, 0.97; 95% CI, 0.66-1.42) with prior SARS-CoV-2 infection.

## Discussion

This population-based cohort study of adults who underwent elective major noncardiac surgery did not find an association of prior SARS-CoV-2 infection with risk of death, MACE, and all-cause rehospitalization within 30 days. These findings were consistent across all subgroups, including among those who were vaccinated and those with the highest estimated operative risk. We did not find that surgery performed in closer proximity to a test positive for SARS-CoV-2 was associated with increased risk of postoperative complications, in contrast to what has been reported previously.^13^ This may be due to inclusion of a more contemporary cohort of vaccinated patients. Alternatively, some of the event rates were low, and the 95% CIs were relatively wide, which does not preclude a possibility of increased risk of outcomes among higher-risk subgroups or with less than 7 weeks between SARS-CoV-2 infection and surgery. Furthermore, it is not clear whether the median time to surgery following a positive COVID-19 test result of 12 weeks represents a delay due to clinical prudence by surgeons to postpone surgery and optimize operative risk or to allow recovery from acute infection with SARS-CoV-2.

To date, more than 550 million people have been infected with SARS-CoV-2, many of whom are awaiting elective surgeries that have been delayed during the COVID-19 pandemic.^1^ The findings from this study may help reassure patients and clinicians that proceeding with surgery after SARS-CoV-2 infection does not appear to be associated with increased risk of adverse operative outcomes. The importance of this may be most applicable to patients awaiting cancer surgery, as recent research suggests that delays in cancer surgery during the pandemic were associated with reduced survival, in which each 4-week delay was associated with approximately 10% greater risk of death.^29,30^

Prior research in patients with preoperative pneumonia and influenza found an association with elevated operative risk and suggested the need to delay surgery for those with infection in the 4 weeks preceding surgery.^31,32,33,34,35,36^ The COVIDSurg Collaborative completed the largest multicenter prospective cohort study to date of 3127 unvaccinated patients undergoing emergency and elective surgery.^13^ That study demonstrated a 4-fold increased odds of death at 30 days in patients with prior SARS-CoV-2 infection compared with those without infection at the time of surgery. These risks appeared to dissipate in patients who underwent surgery more than 7 weeks after SARS-CoV-2 infection.^13^ It is likely that the results from these early studies, which used a positive test result as time 0, altered global clinical practice such that treatment of people with a positive test result in the current study was different and partially explains the observed delay between a positive test result and a person’s surgery. In contrast, we found no association with elevated postoperative risk across multiple outcomes for people with prior SARS-CoV-2 infection regardless of vaccine status, timing of surgery, or estimated preoperative risk. We believe this is because our study included a more contemporary population-based cohort consisting entirely of patients (31% of whom were partially or fully vaccinated) undergoing elective surgery after early pandemic pressures had largely abated and clinical practice may have changed in response to evidence from earlier studies. Our study also reflects contemporary clinical practice, which may include deliberate delaying or postponing of elective surgeries for patients with the highest risk of poor outcomes, including those who had severe COVID-19. Given that we identified no increased risk associated with prior SARS-CoV-2 infection, it is not surprising that COVID-19 vaccination did not significantly modify the risk. This should not be misinterpreted as a sign that vaccination is ineffective. Unvaccinated patients are at higher risk of contracting COVID-19, being hospitalized, or dying after initial infection and, therefore, may have been selected out of candidacy for surgery, biasing the comparison.^37,38,39^

### Strengths and Limitations

A major strength of this study was the inclusion of patients who were vaccinated against COVID-19, which represents an important addition to the limited body of evidence. Other main strengths of our study include (1) assessment of whether vaccination status modified the association of SARS-CoV-2 infection with outcomes, (2) measurement of clinically relevant associated cardiovascular outcomes, (3) stratification by timing of elective surgery relative to SARS-CoV-2 infection, (4) use of a population-based cohort to minimize selection bias, and (5) inclusion of all people who tested negative for SARS-CoV-2 prior to surgery. The latter minimizes the risk of confounding and selection bias due to differential health care seeking behaviors.^15^

This study also has limitations. First, not all people with symptomatic SARS-CoV-2 infection seek medical treatment or testing to confirm their infection, especially if their symptoms are mild. Misclassification resulting from undetected SARS-CoV-2 infection, including among those with a positive test result longer than 6 months before their surgery assigned to the unexposed group, would bias our results toward the null. Still, we found that untested patients who had surgery were similar to those who had testing prior to their surgery, thereby minimizing the potential effects of both misclassification and selection bias in this cohort. Second, we lacked symptom data. Prior research suggests that patients who remain symptomatic even after a 7-week delay, including those with active disease like pneumonia, continue to have increased postoperative risk.^13^ Third, our study period did not include contemporary variants such as Omicron. Recent evidence suggests that infection with Omicron is associated with less severe disease, and therefore, inclusion of data from the period with Omicron would be unlikely to substantially change our results.^40^ Fourth, there is a risk of bias due to unmeasured confounding related to selection of patients for surgery. This potentially important source of confounding bias may derive from unmeasured clinical events or evaluation related to preoperative preparation and management, such as biochemical and hematological screening laboratory testing, that occurred during this interval and may have differed in the period between testing and surgery among those with a positive test result and those with a negative test result. Thus, our results should be interpreted as reflective of current clinical practice, and we have been careful to note the wide 95% CIs for higher-risk subgroups and when surgery occurred less than 7 weeks after SARS-CoV-2 infection. Nevertheless, there was a small absolute differences in outcomes between the 2 study groups. Future work with larger data sets and event rates may consider an analysis matched on time prior to surgery to help address the effects of this potential bias on the outcomes. Fifth, the relatively low number of patients with infection may have limited our statistical power to detect meaningful differences. Sixth, the generalizability of our findings to patients undergoing cardiac and other elective noncardiac surgeries is unknown. Seventh, we focused on cardiac complications and death to address a specific identified knowledge gap; morbidity and mortality in noncardiovascular systems are also important in the discussion of risk among patients and health care practitioners.

## Conclusions

In this study, prior infection with SARS-CoV-2 was not associated with death, MACE, or rehospitalization following elective major noncardiac surgery, although low event rates and wide 95% CIs do not preclude a potentially meaningful increase in overall risk. These findings may have direct applications to health resource planning and utilization as jurisdictions emerge from the pandemic and address massive backlogs in elective surgeries.

## Notes

Supplement 1. **eAppendix.** Description of Datasets
eReferences

Supplement 2.
Data Sharing Statement
